# Weather and Aggressive Behavior among Patients in Psychiatric Hospitals—An Exploratory Study

**DOI:** 10.3390/ijerph17239121

**Published:** 2020-12-07

**Authors:** Jakub Lickiewicz, Katarzyna Piotrowicz, Patricia Paulsen Hughes, Marta Makara-Studzińska

**Affiliations:** 1 Department of Health Psychology, Faculty of Health Sciences, Institute of Nursing and Midwifery, Jagiellonian University Medical College, 31-501 Kraków, Poland; marta.makara-studzinska@uj.edu.pl; 2 Department of Climatology, Institute of Geography and Spatial Management, Jagiellonian University, 30-387 Krakow, Poland; k.piotrowicz@uj.edu.pl; 3 Department of Kinesiology, Applied Health and Recreation, College of Education and Human Sciences, Oklahoma State University, Stillwater, OK 74078, USA; trish.hughes@okstate.edu

**Keywords:** coercion, weather, health personnel, patients

## Abstract

*Background:* The number of meteoropaths, or people negatively affected by weather conditions, is rising dramatically. Meteoropathy is developing rapidly due to ever poorer adaptations of people to changes in weather conditions. Strong weather stimuli may not only exacerbate symptoms in people with diseases of the cardiovascular and respiratory systems but may also induce aggressive behavior. Researchers have shown that patients suffering from mental illnesses are most vulnerable to changes in the weather and postulate a connection between the seasons and aggressive behavior. *Methods*: The goal of the study was to analyze the relationship between coercive measures and weather factors. The researchers identified what meteorological conditions prevailed on days with an increased number of incidents of aggressive behavior leading to the use of physical coercion towards patients in a psychiatric hospital in Poland. In order to determine the impact of weather conditions on the frequency at which physical coercion measures were used, the hospital’s “coercion sheets” from 1 January 2015 to 31 March 2017 were analyzed. The data were correlated with meteorological data. In order to determine the relationship between the occurrence of specific weather conditions and the number of coercive interventions (*N*), researchers utilized Spearman’s rank correlation analysis together with two-dimensional scatter diagrams (dependency models), multiple regression, stepwise regression, frequencies, and conditional probability (%). *Results*: Lower barometric pressure and foehn wind increased aggressive behavior in patients that led to coercive measures. For temperature (positive correlation) and humidity (negative correlation), there was a poor but statistically significant correlation. *Conclusions*: Monitoring weather conditions might be useful in predicting and preventing aggression by patients who are susceptible to weather changes

## 1. Introduction

It is fairly well accepted that weather conditions can impact health, both physiologically and psychologically. The number of meteoropathic people (excessively sensitive to weather) is rising dramatically [1]. It is estimated that in the mid-twentieth century, every third person reacted to weather changes; currently, this group represents about half of the population. Meteoropathy is expanding rapidly due to an ever poorer adaptation of people to changes in weather conditions, which is exacerbated by little outdoor time and spending the majority of time in climate-controlled environments.

Strong weather stimuli may exacerbate symptoms in people with mental illnesses [1,2,3,4]. At such times, people sensitive to weather conditions may experience headaches and dizziness [5,6], changes in blood pressure [7], aggression [8], and accelerated heart rates [4]. Researchers in different countries have determined that patients suffering from anxiety [9], depression [3], schizophrenia [10,11], and bipolar disorder [12,13,14] are particularly vulnerable to changes in the weather. McWilliams et al. saw a correlation between barometric pressure and internal hospital admissions for mania [15]. 

In Canada, the number of patients presenting themselves to emergency services for mental and psychosocial problems is higher at times of increased temperature and humidity [16]. According to Brandl et al., there were more ER patients in a psychiatric hospital on warm and cloudy days [17]. In Israel, there were also more psychiatric admissions for patients with schizophrenia during the hottest weather [18]. According to a Vida et al., (2012) study, average temperature accounted for 3.5% of total variance in the number of patient admissions at mental health emergency departments in Québec, Canada [16]. The daily number of patients consulting the emergency room (ER) of a psychiatric hospital in Germany was significantly higher during spring and summer, compared to fall and winter [17]. 

In one of the first studies of its kind, Semenza (1996) found that mental health conditions were more life-threatening during a heatwave than any other chronic health condition, including cardiovascular and respiratory conditions [19]. Bouchama (2007) later found similar results and stated that psychiatric illness tripled the risk of death during heat waves [20]. During heat waves, emergency rooms see more psychiatric patients [21]. For those with mental illnesses, extreme heat can interact with medications and impair the ability to regulate body temperature; thus, extreme heat poses physiological issues that may be life-threatening for such people [22,23]. Extreme heat, for instance, is known to lead to an increase in suicidal behavior [24] and violence [25]. Lõhmus (2018), in a recent review of the literature, concluded that although high temperatures do not cause mental illness, they likely worsen existing conditions [26].

Other researchers have failed to find climate associations with behavior. In regard to bipolar disorder, neither latitude nor climate were associated with mood changes among patients [27].

Researchers point to a connection between aggression in clinical settings and hospitalization that are directly related to air temperature, relative humidity, and/or daily atmospheric pressure [28]. More psychiatric visits occurred in Spain by patients with psychiatric histories and more inwardly and outwardly violent behavior, and who required greater use of restraints during hot temperatures, but no differences in the number of psychiatric emergencies or new admissions in psychiatry emergency departments were found [29]. In Finland, the least aggressive behavior was found among patients in winter [30].

Meteorotropic factors include rapid weather changes associated with, among other things, the passage of weather fronts, changes in the type of air masses, storms, and strong wind, including foehn, which is typical in foothill areas [4]. The circulation type, movement of specific air masses, and atmospheric fronts determine the daily course of various meteorological elements, which are known to have a strong stimulating effect on the human body [31].

In each region, weather can have a different impact. For example, in Syria, episodes of schizophrenia appear more frequently in May or June, and in Bosnia and Herzegovina, hospital admissions of neurotic, somatoform, and stress-induced disorders are observed to vary across months [32]. According to some researchers, female patients appear to be more sensitive to the effects of weather than men [13,33]. Researches in Finland found a drop in the use of coercion in January [22], and an increase between June and November [34]. The relationship between weather and aggression was also revealed for auto-aggressive behaviors, the most extreme form of which is suicide [35,36]. In Finland, suicidal attempts by men were most frequent during low atmospheric pressure, while women’s attempts occurred most often when atmospheric pressure was high [37]. 

Weather that impacts behavior includes rapid changes associated with the passage of weather fronts, type of air masses, storms, and strong wind, including foehn wind, which is characteristic of foothill areas [4]. Such a wind is known in the Alps as föhn (same as foehn), as Chinook in the Rocky Mountains (U.S.), Zonda in the Andes, and Halny in Poland. Its impact on the human body, health, and well-being has been studied by several researchers [4,5,6,7]. There is a widespread view that foehn situations have a strong impact on the nervous system [4,38].

Related literature also points to staff-related factors, such as the role of the ward, age, and clinical experience of the medical professionals as triggers, along with personality determinants of staff and patients alike. The perception of aggression and attitudes towards it are also important, as well as rules in the ward, and even its architecture [39].

Aggressive patient behavior poses a substantive challenge for medical personnel. The triggers of such behavior include a number of interrelated elements, including, but not limited to, traits of patients and visitors, their emotions, and health [39,40]. Most patients are non-aggressive and their behavior is easily managed. For some, however, behavior is volatile and often aggressive. For such behaviors, staff must have options for control to ensure the safety of all. In extreme instances, physical restraint of the patient must be used to ensure the safety of all. In healthcare, the concept of “coercion” is usually associated with psychiatric patients for whom physical restraint in treatment remains the last option [41,42]. Coercive measures are used to control patients, or, depending on the situation, overpower and immobilize them completely to ensure safety. Medical staff can resort to a number of measures in response to incidents involving aggressive behavior by patients. However, the use of physical coercion towards patients in psychiatric hospitals poses a major systemic and ethical dilemma, as depriving patients of the right to self-determination causes strong anxiety and a perception of threat, both to givers and receivers of the coercion [43]. 

Use of coercion to control patient behavior is allowable under four circumstances in Poland: an attempt on one’s own or another person’s life or health, an act against public safety, violent destruction or damage to objects in the patient’s surroundings, or disruption to or prevention of the operation of a healthcare institution. Physical coercion is only employed in situations when aggressive behavior is intended to inflict suffering or cause damage. The use of coercive measures is regulated by the Mental Health Act, according to which acceptable coercive measures include holding, drugs, mechanical restraints, and seclusion. 

The most commonly used means includes mechanical restraint, which should be documented in accordance with the Polish legislation. The document used for this purpose, the “coercion sheet”, states the reason, the beginning and ending time, and the persons involved. According to the Mental Health Act, coercive measures should be selected in such a way as to be the least restrictive to the patient, and the patient’s well-being should be given priority. Further, physical coercion can only be used until the reason justifying it ceases to exist [44].

Thus far, there have been few reported studies in Central Europe exploring the relationship between weather conditions and aggressive behaviors among patients in psychiatric care. In a number of biometeorology studies, when analyzing the impact of weather conditions on various types of disorders, investigators have taken into account individual meteorological elements or biometeorological indicators that combined several factors simultaneously [1,2,14,15,16,37,38,39,40,45,46,47].

There is a gap in knowledge about the relationship between meteorological conditions and using coercive measures. This issue is important from a patient’s safety perspective. Although extreme heat is a known stressor to the body, Lõhmus (2018) suggested that we know more about the effects of heat on the body than we do about other conditions, such as humidity, wind, or barometric pressure [26].

Therefore, the first purpose of the study was to determine the prevailing meteorological conditions in addition to heat, on days with a high number of aggressive behaviors leading to the use of physical coercion with patients. A second purpose was to identify discrete weather components, as well as multiple components acting together that accompanied use of coercive restraints. Recognizing relevant weather elements that precede aggressive behavior may be helpful in preventing patients’ pathological responses, or failing that, preparing hospital staff for a likely upturn in behaviors requiring coercion.

## 2. Materials and Methods

### 2.1. Medical Data

This paper is part of a research project to evaluate the effectiveness of tools for analyzing aggressive behavior in psychiatric settings and was approved by the Bioethical Committee of the Collegium Medicum at the Jagiellonian University (No. 122.6120.322.2016), and funded by the Ministry of Science and Higher Education of Poland (K/ZDS/006182). To determine the impact of weather conditions on the use of physical coercion, the researchers analyzed the hospital’s coercion sheets from January 2015 to March 2017. Researchers classified events for further analysis if intervention involved compulsory drug administration, mechanical restraints, or isolation, and disregarded solely verbal aggression. Researchers noted the date when a coercive measure was resorted to, which meant that aggressive behavior had occurred shortly beforehand. The study focused on days with a high number of coercive interventions. The level adopted as “high” was more than 8 *(N* > 8) and resulted from the frequency distribution (from 1 to 19 cases that occurred each day) and a value that exceeded the mean number of coercive interventions per day (mean and median = 8).

### 2.2. Meteorological Data 

Meteorological conditions for each day of the 27-month study period were obtained from the climatological station of the Jagiellonian University in Krakow. The authors included two years of winter months in the study (December–February) because, from a climatological perspective, the volatility of meteorological conditions in Central Europe is at its highest. Data included air temperature (mean, maximum, and minimum; °C), air pressure (hPa), relative humidity (%), cloudiness (%), sunshine duration (h), and precipitation (mm), along with wind speed (m/s) and direction. In order to determine the cumulative impact of weather on the number of coercive interventions, the researchers used the Synoptic Situations Calendar prepared by Niedźwiedź [48], which can be used to identify types of circulation over southern Poland, the direction of horizontal air mass movement, and type of air mass, as well as the presence of weather fronts. 

Weather conditions affecting health include low pressure systems, passage of weather fronts (especially cold ones), movement of air masses with contrasting physical characteristics, stormy weather (unstable atmosphere), and foehn situations (foehn—a dry and warm wind blowing from the mountains). A foehn wind, which is caused by a rise in air temperature, a drop in relative humidity, and a strong wind at the foot of mountains about 100 km from Krakow is a normal occurrence in Krakow. Only those cases in which foehn lasted at least 6 h during daylight hours were included in the analysis, because of the distance between Krakow and Zakopane. Taking into account types of circulation, air masses, weather fronts, and foehn situations enabled the researchers to consider the cumulative influence of various meteorological elements, and not just each condition separately. The researchers analyzed separately the conditional probability of the occurrence of days with an increased number of coercive interventions at times of foehn wind. 

### 2.3. Statistical Analyses

In order to determine the relationship between the occurrence of specific weather conditions and the number of coercive interventions (*N*), including an increased number (*N* > 8), researchers utilized Spearman’s rank correlation analysis together with two-dimensional scatter diagrams (dependency models), multiple regression, stepwise regression, frequencies, and conditional probability (%). 

In several cases, large and highly statistically significant values of the correlation coefficient were of no value due to their lack of predictive validity. Therefore, the total influence of selected meteorological elements was also analyzed with the use of a multiple regression model. Since the model should comprise weather conditions strongly correlated with a high number of coercive interventions and at the same time intercorrelated as weakly as possible, a stepwise regression model was used (forward and backward). The coefficient of correlation (R) was used to assess the goodness of fit of the models. The R value represents to what extent the estimated model explains the original variance of the values of the dependent variable and how much of the variance is random or can be explained by the effect of other independent variables not included in the model [49]. Such regression models allow a better understanding of the variables under study, and when they demonstrate a good fit, they can be used to predict or simulate values of the dependent variable at specific values of the independent variables [50]. Simultaneously with correlation analyses, simple descriptive methods were also used in the study, including conditional probability, based on the joint influence of several meteorological elements which together form a certain type of condition or occur during the foehn wind.

### 2.4. Study Location

The study involved data from the largest psychiatric hospital in the Lesser Poland Province—Specialist Hospital Dr. Joseph Babinski in Krakow. The hospital can accommodate 790 patients, with 17 inpatient wards, 6 day care wards, 9 community mental health treatment teams, and 6 outpatient clinics. 

In this hospital, four categories of patients are treated: addiction, forensic (low and medium security), general psychiatry, and geriatrics, annually providing approximately 8500 inpatient services and around 85,000 outpatient and day care services. For study purposes, only data from inpatient wards were analyzed. During the research period (January 2015 to March 2017), a total of 17,974 were inpatients, with 7431 in 2015, 8044 in 2016, and 2589 in 2017.

The region lies in southern Poland and has an area of 15.2 thousand km^2^ inhabited by 3.4 million people (2016), and of which Krakow is the capital. The average annual temperature of the area is 9.3 °C/48.7° F (data in the period 2001–2010). Usually, July is the hottest (20.4 °C/ 68.7° F) and January is the coldest month (−1.6 °C/32° F). Due to its location at the bottom of the Vistula River valley, weak westerly winds prevail in the area (<2 m/s). The cold half of the year (November–April) is under the influence of a foehn wind originating in the Tatras Mountains to the south of Krakow.

## 3. Results

### 3.1. Days with Coercive Interventions among Hospital Patients

The analysis included days with all coercive interventions (*N*) as well as days with an increased number (*N* > 8) in the period from 1 January 2015 to 31 March 2017. There were 329 days (out of 820 possible) with *N* > 8, out of which 24.5% and 28.4% were days with 9 or 10 coercive events, with the greatest number (*n* = 19) recorded on 10 July 2016.

Given the annual variability of meteorological conditions in the moderate climate of Central Europe, the researchers determined the part of the year (seasons, months) with the greatest number of interventions (*N* > 8). Such days were slightly more frequent in the cold half of the year (November–April), especially December through February. In 2016, quite a large number of them also occurred in spring (March–May) and autumn (September–November). However, when analyzing the number of days with *N* > 8 by months, the highest number occurred in April 2016 (20 days), while the lowest was also in April, but of 2015 (two days). Thus, based on the available data, the researchers identified fewer coercive measures used during the summer (June, July, and August). 

### 3.2. Meteorological Conditions on Days with High Number of Physical Coercion Events

Rather than occurring separately, sometimes days formed long strings. Even though such cases represented 60% of the days with *N* > 8, about one third of the strings lasted two, three, or four days. The case in which physical coercion was used with at least nine patients for 13 successive days occurred between 14 November and 26 November 2016, and was analyzed separately.

When analyzing the impact of weather components on various types of conditions, individual meteorological elements, or biometeorological indicators, combining several factors simultaneously is typically utilized by researchers [2,4,16,45,47]. 

First, the values of Spearman’s rank correlation coefficient between *N* and *N* > 8, individual meteorological elements, and two-dimensional scatterplots were used. Statistically significant (*p* < 0.05) relationships occurred for *N* > 8—air masses, mean and maximum air temperature, and also barometric pressure (Table 1). The number of days with more frequent coercive interventions increased with a rise in temperature (positive but poor), and increased with a drop in air pressure (negative correlation). In various regions of the world, extreme heat events lead to an increase in the number of patient presentations to hospitals due to mental illness [51,52,53], but in our study, where patients were kept in closed hospital wards, the effect seemed to be much smaller, statistically bordering significance. Moreover, the short series of data (only two summer seasons) does not allow for a more precise analysis of the relationship between the number of hot days and the aggressive behavior of patients.

Further analysis took into account the joint influence of various meteorological elements.

The application of the multiple regression model did not produce statistically significant correlations (*p* > 0.05) between the analyzed events and the eight meteorological elements. Independent variables for the model were sought using stepwise regression (forward and backward). However, examination of more than a dozen models did not provide grounds for accepting any of them. It seemed that a better solution was to take into account such indicators or types of weather, which together comprise a high number of variables. Therefore, an analysis of the total meteorological situation and the impact of the foehn wind on the examined phenomena was carried out as a next step.

A slightly higher conditional probability of the occurrence of days with *N* > 8 was found for low atmospheric pressure (43.1%) than for high atmospheric pressure (37.9%). Probability of over 50% occurred with movement of air masses from the north for low pressure (64.3%) and northwest for low (61.8%) or high pressure (51.7%). Therefore, with these three types of circulation, a rise in the frequency of days with a high number of coercive situations can be expected.

A similar analysis of the correlation between *N* > 8 and the accompanying types of circulation was conducted for air masses and weather fronts moving over Krakow. Even though the results did not demonstrate clearly which air masses predominated on the days with *N* > 8, it must be emphasized that the passage of multiple air masses over a given area during the day is indicative of rapid weather changes and that such meteorological conditions can strongly stimulate the body [17,36]. 

In regard to fronts, days with *N* > 8 were correlated with the passage of a cold front (46.0%) or several fronts on one day (45.6%). These two cases are associated with dynamic changes in the weather, which can have an irritating effect on humans [9,28,47]. 

### 3.3. Occurrence of Foehn on Days with an Increased Number of Aggressive Incidents

The researchers identified 123 days of the 820 total days with a foehn wind. The wind is the most frequent in the cold half of the year (November–April), therefore the frequency of its occurrence and the corresponding days with a high number of coercive interventions (*N* > 8) were analyzed by individual months. In the colder half of the year, the conditional probability of the events analyzed was 50% in February and April 2015 and 55% in February 2016, with the maximum probability of 58% in November 2016. However, it should be noted that the potential conditions for the occurrence of foehn in the Tatra Mountains included in the analysis comprised cases when the wind continued for at least six hours during the day. The effects of such a short-lived phenomenon may not have been felt in Krakow, which is about 100 km away from the mountains. Nevertheless, the effects of foehn on aggressive behavior involving coercion towards patients in Krakow can be traced on the basis of the example of the longest string of days with coercive situations, which took place between 14 and 26 November 2016.

### 3.4. The Longest String of Days with Coercive Interventions and Associated Meteorological Conditions (14–26 November 2016)

The analysis of meteorological data shows that air temperature in southern Poland was high for the season (>10 °C/50 °F) between 14 November and 26 November 2016, which was indicative of a foehn wind in the mountains. Foehn started on 15 November, but an increased number of incidents of aggressive behavior, which were associated with the gradual change in atmospheric pressure, air temperature, and relative humidity in Krakow, had already begun the preceding day. On subsequent days, the wind determined the weather in southern Poland with varying intensity until 24 November. However, an increased number of incidents with aggressive behavior leading to the use of physical coercion continued to be seen for two more days after the foehn ended. Its adverse effect was manifested both by high winds velocity (in Krakow, the average hourly values were 2.5–5.7 m/s, with a maximum speed of 10–15 m/s) and its pulsating character (pseudo-calms and violent gusts), as well as by its variability in the breadth of meteorological factors accompanying the phenomenon (including an increase in temperature and decrease in humidity).

## 4. Discussion

The purpose of the study was to conduct initial research in Poland in regard to the relationship between weather conditions and coercive measures used with patients in psychiatric care. The researchers recorded prevailing meteorological conditions and attempted to identify meteorological commonalities on days with a higher than average number of aggressive behaviors leading to the use of physical coercion. By identifying weather types that preceded aggressive behavior, the authors speculated that there would be some predictive information for aggression that would be useful to medical staff to prepare them for a likely upturn in behaviors requiring coercion.

Researchers typically have focused on the impact of individual meteorological elements or biometeorological indicators on various types of mental disorders or their seasonal variations [2,16,30,45]. Few researchers studying diseases of the nervous system have investigated the cumulative effect of elements over a given area (pressure systems with directions of specific air masses and the presence of weather fronts). A cumulative effect of weather elements can be analyzed on the basis of the types of circulation, air masses, and weather fronts prevailing on a given day and in a given area. An analysis of medical data leads to the conclusion that an increase in the use of mechanical restraints is accompanied by:

(1) Low-pressure systems, with winds from the north or northwest;
(2) The passage of various air masses during one day and/or movement of cold or Arctic air masses, as rapid changes in temperature are more likely to be problematic than steady temperatures;
(3) A drop/change in barometric pressure precluding a storm;
(4) A foehn wind, especially when it lasts for a longer spell (at least a few days) and is intense (with a sudden change in the values of individual meteorological elements and high winds with strong gusts).

However, it should be noted that in other regions of the world, an increase in aggressive behavior may be associated with the occurrence of a different type of regional wind. For example, in the semi-arid areas of Israel, the eastern desert wind from the Saudi Arabian, Libyan, and Sinai deserts contribute to an increase in the likelihood of psychotic relapse and suicide attempts, while a westerly sea breeze does not [36,54].

Air temperature is also an important variable for people with mental illnesses. The effect of heat waves was identified in a temperate Australian city for mental, behavioral, and cognitive disorders [55]. The authors observed an increase of 7.3% between ambient temperature above a threshold of 26.7 °C and hospital admissions which included organic illnesses, including symptomatic mental disorders, mood (affective) disorders, neurotic, stress-related, and disorders of psychological development. Hansen et al. (2008) suggested that heatwaves pose a salient risk to the health and well-being of the mentally ill [55]. Sanz-Barbero and Linares et al., (2018) reported that the risk of interpersonal violence increased three days following a heat wave and calls to a violence helpline increased five days after the heat wave. In the current study, the researchers did not note such a phenomenon [56]. Arifwidodoa and Chandrasirib (2020) found that urban heat stress significantly affected human health, including mental health. Thus, the increased risk of heat stress represents a major problem in urban areas in relation to climate change. These effects are likely to intensify in the future [51]. Mullins and White (2019) also found that higher temperatures increased emergency department visits for mental illness and suicides. Cold temperatures, on the other hand, reduced negative mental health effects [57]. The direct and indirect impacts of high indoor temperatures on physical or mental health were identified in a study by Tham et al., (2020). The authors concluded that symptoms of schizophrenia and dementia were significantly exacerbated by indoor heat (indoor temperature threshold above 26 °C). Worsening of symptoms in warm indoor environments is important for both patients and those who care for them. In their management plans, care staff and facility managers need to anticipate the possibility of an increased risk of mental illnesses symptoms in overheated rooms. Unfortunately, the authors of this study did not have access to the temperature within the hospital. Further, the analysis of only two summer seasons did not allow for a detailed analysis of the impact of heat waves on the number of aggressive behaviors [51].

Practical applications for this study include several key points. Knowing weather conditions preceding an increased number of aggressive incidents (the weather situations mentioned above and foehn wind) allows staff members to put preventive measures in place, which are much more effective than reactive ones [58].

## 5. Conclusions

Information about weather components on a given day and those expected over the next few days is widely available, since it serves as a basis of weather forecasts. Medical staff can begin to monitor weather data, including temperature, barometric pressure, and wind direction, and thus begin to compile a profile of types of weather conditions before an increased number of aggressive incidents. A number of mobile weather applications are available for free or low cost that easily yield needed information. Barometric pressure appears to be generally on different applications than those yielding temperature and wind direction and velocity. Although not all cell phones have barometric sensors, there are a number of applications available for monitoring barometric pressure. At least one application yields an alert when the barometric pressure changes, enabling medical staff to predict an upturn in aggressive incidents and intercede with interventions known to be predictably calming for patients prone to aggression/violence. 

This study possesses some limitations. Firstly, aggressive incidents requiring physical restraint of the most aggressive type were analyzed. The criteria for use of restraints might vary from place to place. Secondly, data analyzed were from one site only, although the authors had a large sample size across 27 months. There is no way to know if the data are generalizable across all of Poland, or across continents. Third, data were not analyzed by condition or sex. Fourth, the investigators did not differentiate between foehn winds lasting 6 h versus those lasting much longer. Further effects may have been distinguishable. Fifth, psychiatric disorders were viewed together and not examined separately. Previous researchers have pointed to disorder-specific weather conditions. A weather condition that might affect a meteoropath with bipolar disorder is not necessarily the same weather condition that would impact a person with depression [59]. Last, the research is retrospective, so the relationship between weather variables and physical coercion should be seen as associative, not causative. 

Three things can be accomplished by recording weather data. Firstly, staff can begin to predict when aggressive behavior is likely to occur, and secondly, staff can identify which patients are particularly meteoropathic. Thirdly, staff can begin to build a profile of which weather conditions seem to be associated with aggressive behavior in that particular locale and/or with specific patients.

It would be interesting for future research to analyze other factors such as blood pressure and heart rate, which might affect patients’ behavior, regardless of weather variables. As mentioned before, weather conditions, like heat, affect patients and medical personnel alike. It might be beneficial in future research to analyze the influence of those factors on medical personnel behavior.

Preventing aggressive behavior is a goal for all medical personnel working with psychiatric patients. The overarching objective is to prevent aggressive behavior rather than intervene after it has already occurred. The authors provide a tool to give staff some foreknowledge of things likely to occur. With that knowledge, safety may be enhanced for all.

## Figures and Tables

**Table 1:** Results of Spearman’s rank test examining the association between coercive interventions among hospital patients (*N*—days with all coercive interventions, *N* > 8—days with nine or more, *—*p* < 0.05) and meteorological parameters in Krakow.

Meteorological Elements	*N*	*N* > 8
Types of circulation	0.089 *	0.084
Air mass	0.047	0.126 *
Weather fronts	0.037	0.027
Air pressure (hPA)	−0.073 *	−0.109 *
Maximum air temperature (°C)	0.036	0.110 *
Minimum air temperature (°C)	0.070 *	0.102
Mean air temperature (°C)	0.056	0.109 *
Relative humidity (%)	0.037	−0.024
Wind speed (m/s)	0.046	0.005
Cloudiness (%)	0.018	0.047
Sunshine duration (h)	−0.023	−0.004
